# The Importance of Peer-Reviewers. How to Recognize their Merits?

**DOI:** 10.21470/1678-9741-2017-0054

**Published:** 2017

**Authors:** Domingo M. Braile

**Affiliations:** 1 BJCVS

After restructuring the editorial process of the Brazilian Journal of Cardiovascular Surgery (BJCVS) in 2016, rewarding the good reviewers for their excellent performance is a goal set by the publication team.

The reviewers are the ones who are responsible for spreading the valorization and the qualification of our scientific publications in the national and international scope.

In an article published in Nature^[1]^ in 2014, the author expresses concern about the sustainability and quality of the peer review system, a fact proven by his own experience and his colleagues when receiving inadequate opinions, showing a lack of understanding of the content by the reviewer. The increasing number of online journals in recent decades has been contributing to reviewers’ overload. In addition to that, the editorial policy of some open access journals is to publish articles whose methodology is valid and which “has its accuracy and validity certified, regardless of the evaluation of its content.” This recommendation may sound to reviewers as a signal to approve articles only based on analysis of methods and statistics sections, without considering the reasons why it was conducted and its broader context.

According to Lilian Nassi-Calò^[2]^, a Scientific Electronic Library Online (SciELO) collaborator, “Peer evaluation has been going through a transition period and many believe it is necessary to redefine its principles and practices in order not to delay or impede the scientific progress. In this regard, alternatives to the traditional arbitration model have recently emerged, which is usually closed, maintaining the identity of the reviewers (single-blind system) and also the authors (double-blind system) confidential. It is interesting to point out that since 1833 closed and anonymous opinions prevailed, since it was not considered appropriate or cordial to publicly review a colleague’s work, moreover, opinions were not considered a personal expression, but represented the author’s hierarchy.

There are several models to reward the reviewers, but the most cogitated one in recent times proposes the public recognition of the evaluators to give prestige to this important stage of the publishing process of scientific publication and, consequently help improve the quality of revisions.

The BJCVS, with the intention of honoring its reviewers, joined the [Publons], a website that was created to record the input of peer reviewers and influence researchers to offer their experiences as reviewers.

According to an online interview, see below the opinion of Andrew Preston^[3]^, co-founder of [Publons]. All reviewers should sign up at [Publons] to register their revisions both before and after the online publication. The manuscript, prior and after to the reviews, is not disclosed, unless allowed by the journal. However, we request the authors the review certificate or we check with the editors to make sure the review was performed by the one selected for such task. To increase our performance, we are working to automate the system, speeding up the update of all reviewers. Even when a journal opts for anonymous reviews, our platform may give credit to reviewers. It’s easily understood that when you are selected as reviewer of an article, the publisher is demonstrating that you are considered a specialist in that subject. [Publons] is a great way to highlight this experience. Our users are beginning to use their certificates as reviewers on their curriculums, and also to be recommended for the journals’ boards, or selected as associate or full editors.

Predicting that the reviewer’s activity would be one day recognized, the BJCVS has been issuing certificates for each review carried out by its collaborators since 2002.

These data are also filed in our databases, and can be obtained by their request.

In order to make the reviewer’s activity more recognized, we have long been claiming that “CAPES - Coordination for the Improvement of Higher Education Personnel in Brazil” should consider this procedure of high relevance, essential to guide authors in correcting flaws, either in form or content, increasing the quality of journal’s articles, fundamental measure for the research’s be cited, resulting in the increase of the Impact Factor, placing our country in the spotlight of the Concert of Nations. One day, we hope to be able to obtain this benefit by valuing the revisions as if they were “scientific articles”. The [Publons] may represent a new argument with the CAPES leaders, so that our idea can be accepted. For this reason and for the benefit it represents in curriculum content, we recommend that all associated editors and reviewers of the BJCVS, as well as the authors, who will at some point be invited to be reviewers, sign up for [Publons] by accessing the link: https://Publons.com/home/.

I hope that reading this first issue of 2017 will be interesting for all who can find in our journal a source of knowledge and incentive to promote it nationally and internationally.

Warmest regards!

**Domingo M. Braile** ^1^Editor-in-Chief - BJCVS

## References

[1] Arns M (2014). Open access is tiring out peer reviewers. Nature.

[2] Nassi-Calò L (2017). Aumenta a adoção de avaliação por pares
aberta.

[3] Van Noorden R (2014). The scientists who get credit for peer review. Nature.

